# Validity, reliability, and sensitivity to motor impairment severity of a multi-touch app designed to assess hand mobility, coordination, and function after stroke

**DOI:** 10.1186/s12984-021-00865-9

**Published:** 2021-04-23

**Authors:** Sara Mollà-Casanova, Roberto Llorens, Adrián Borrego, Bárbara Salinas-Martínez, Pilar Serra-Añó

**Affiliations:** 1 UBIC, Departament de Fisioteràpia, Universitat de València, Valencia, Spain; 2 Neurorehabilitation and Brain Research Group, Instituto de Investigación e Innovación en Bioingeniería, Universitat Politècnica de València, Valencia, Spain; 3 NEURORHB. Servicio de Neurorrehabilitación de Hospitales Vithas, Fundación Vithas, Valencia, Spain; 4 Neurorehabilitation and Brain Research Group, i3B Institute, Universitat Politècnica de València, Ciudad Politécnica de la Innovación, Building 8B, Access M, Floor 0. Camino de Vera s/n, 46022 Valencia, Spain

**Keywords:** Hand, Upper-limb, Assessment, Stroke, Multi-touch technology, App

## Abstract

**Background:**

The assessment of upper-limb motor impairments after stroke is usually performed using clinical scales and tests, which may lack accuracy and specificity and be biased. Although some instruments exist that are capable of evaluating hand functions and grasping during functional tasks, hand mobility and dexterity are generally either not specifically considered during clinical assessments or these examinations lack accuracy. This study aimed to determine the convergent validity, reliability, and sensitivity to impairment severity after a stroke of a dedicated, multi-touch app, named the *Hand Assessment Test*.

**Methods:**

The hand mobility, coordination, and function of 88 individuals with stroke were assessed using the app, and their upper-limb functions were assessed using the *Fugl-Meyer Assessment for Upper Extremity*, the *Jebsen-Taylor Hand Function Test*, the *Box and Block Test,* and the *Nine Hole Peg Test*. Twenty-three participants were further considered to investigate inter- and intra-rater reliability, standard error of measurement, and the minimal detectable change threshold of the app. Finally, participants were categorized according to motor impairment severity and the sensitivity of the app relative to these classifications was investigated.

**Results:**

Significant correlations, of variable strengths, were found between the measurements performed by the app and the clinical scales and tests. Variable reliability, ranging from moderate to excellent, was found for all app measurements. Exercises that involved tapping and maximum finger-pincer grasp were sensitive to motor impairment severity.

**Conclusions:**

The convergent validity, reliability, and sensitivity to motor impairment severity of the app, especially of those exercises that involved tapping and the maximum extension of the fingers, together with the widespread availability of the app, could support the use of this and similar apps to complement conventional clinical assessments of hand function after stroke.

**Supplementary Information:**

The online version contains supplementary material available at 10.1186/s12984-021-00865-9.

## Background

Approximately 80% of stroke survivors suffer from motor dysfunctions that affect one or both upper limbs, with particular impacts on hand coordination and dexterity [1, 2]. Hand and upper-limb impairments are among the major causes of functional limitations in individuals with post-stroke hemiparesis [2, 3]. These limitations can reduce autonomy and, therefore, have consequences for the performance of daily living activities and decrease quality of life [3].

An adequate assessment of all motor impairments is necessary for establishing a realistic prognosis, planning customized rehabilitation interventions, and evaluating the effectiveness of those interventions. The assessment of upper-limb motor function is especially challenging because of the multidimensional nature of coordinated movements, requiring the use of multiple subsystems: eye-hand coordination, intra-limb coordination (including inter- and intra-muscle coordination), and inter-limb coordination [1, 4, 5]. In the clinical setting, assessments of motor conditions are usually performed using ‘standardized’ clinical scales and tests [6]. Most clinical scales evaluate the active range of upper-limb movements [7], gross [8, 9] or fine arm motor function [8, 10], and the performance of daily functional activities [11–14], with some scales aiming to assess hand motor function and grasping during functional tasks [8, 15, 16]. Although these tools are usually easy to administer and are not time-consuming, instruments that are based on subjective ratings of the performance on different tasks may lack accuracy and be biased. In addition, instruments that are rated according to the performance on a task (such as the number of elements that can be grasped, moved, or placed or the time to complete those actions) may lack specificity, and not provide separate information of the different motor components that contribute to the performance. In addition, conventional instruments do not commonly consider hand mobility and dexterity during clinical assessments, and when they are considered, the examinations often lack specificity. Importantly, these skills are necessary to perform fast selective wrist and finger movements (wrist-finger speed) and to manipulate small objects (finger dexterity) or larger objects (manual dexterity) efficiently [17], and require precise thumb and finger movements, which can be severely reduced after stroke [18]. Finally, the ability to keep the arm steady (steadiness), to move it quickly and precisely to an intended target (aiming), or to move it under constant visual control along a line (tracking) are equally relevant to arm motor function [17] and difficult to quantify.

Different technological solutions have been suggested to register the complex anatomy and mechanics of the hand [19], mostly using robotic devices [20–23], gloves [24–26], and camera-based solutions [27, 28]. Although these devices have primarily been used for rehabilitation, they also have the potential to overcome the limitations associated with conventional clinical tools, by providing objective and accurate measurements and performing separate analyses of specific finger movements [23]. Unfortunately, many of these systems are either expensive and require dedicated space in the clinic or are not widely available, which limits their clinical use. Multi-touch technology, such as that included in many current smartphones and tablets, allows for the very precise detection of finger touches and hand gestures on a capacitive screen [29]. This feature, together with the portability and low-cost features, could enable the successful assessment of hand mobility and dexterity with tablet devices and facilitate their use not only in the clinical setting but also at home. Previous research examining the use of tablet apps by persons with stroke has shown that interactions with these tools are feasible and acceptable [30], with most individuals being able to perform basic gestures on a tablet with at least one hand [2, 31], whereas the level of participation is dependent on the motor impairment severity [2]. However, only two studies have investigated till date the feasibility of tablet-based assessments of the upper limb motor function after stroke. These preliminary studies showed excellent discriminative validity of different exercises, which included tapping [2, 32], and drawing and coordination exercises [32]. These later exercises also showed poor to good reliability [32]. Although these studies showed the potential of tablet apps to assess motor impairments after stroke, they include scarce exercises, ranging from one to three, and enrol a limited number of stroke survivors or mixed neurological conditions.

We have designed a free, dedicated app to examine hand mobility, coordination, and function, by measuring performance on a series of exercises that attempt to represent the hand movements associated with daily basic activities [33, 34], including tapping, the analytic extension of the fingers, pincer grips with different fingers, hand opening and closing, and visuomotor coordination during drawing and target reaching^1^ [35]. We hypothesized that the proposed exercises had convergent validity with clinical instruments, and were reliable and sensitive to impairment severity. If these hypotheses were corroborated, the multi-touch exercises could complement clinical assessment of the arm and hand function with more objective and accurate measures of specific finger and hand movements.

Consequently, the objectives of this study were three-fold. First, to determine the convergent validity of the app compared with standardized clinical tests performed in a representative sample of individuals with stroke. Second, to quantify the reliability of the app, as defined by inter- and intra-rater reliability, standard error of measurement, and minimal detectable change. Finally, to investigate the sensitivity of the app for the differentiation of post-stroke motor impairment severity.

## Methods

### Participants

Participants with stroke were recruited from the outpatient services of Vithas Hospitals Valencia al Mar and El Consuelo (València, Spain), Aguas Vivas (Carcaixent, Spain) and the Brain Injury Center of Vithas Vinalopó (Elx, Spain). All participants were participating in a long-term rehabilitation program, customized to their particular needs. The assessment period was from May 2018 to September 2019.

The inclusion criteria for participation in the study were as follows: (1) diagnosis of ischemic or hemorrhagic stroke, by computed tomography or magnetic resonance imaging; (2) active movement of distal joints, defined as scores above 1 on the Medical Research Council Scale for Muscle [36]; and (3) fairly good cognitive condition, defined as scores above 23 on the Mini-Mental State Examination [37]. The exclusion criteria were as follows: (1) severe hypertonia, defined as scores below 3 on the Modified Ashworth Scale [38]; (2) impaired comprehension that hindered the ability to follow instructions, defined as scores below 45 on the Mississippi Aphasia Screening Test [39]; (3) severe visual or auditory deficits that prevented interactions with the app; and (4) unilateral spatial neglect.

A minimum sample size of 88 participants was required to ensure a power of 0.80, assuming a medium effect size of 0.3 and an error probability of 0.05, during the analysis of convergent validity. An additional 10% of the sample size was also considered necessary to account for potential data loss.

Ethical approval for the study was granted by the Ethics Committee of Universitat Politècnica de València (P10100120). All eligible candidates who agreed to take part in the study provided written informed consent.

### Instrumentation

A 12″ tablet, the Chuwi Hi12 (Chuwi Technology, Shenzhen, China), which incorporated an Intel Quad Core × 5-Z8300 and 4 GB RAM, was used to run the *Hand Assessment Test* [35], a dedicated app that assesses hand mobility, coordination, dexterity, and functionality. Specifically, the app includes 6 tests. Four tests, the finger tapping, tapping with each finger while the remaining digits are weight-bearing, pincer grasps with all of the fingers, and hand opening and closing, evaluate the body functions and structure [40]. The remaining two tests, graphomotricity and oculo-manual coordination evaluate the activity [40]. All the exercises work equally. They all start with a three-second countdown that leads to an auditory signal that indicates the start of the test. The end of the test is also indicated with an auditory signal. After the test, the exercises provide the outcome measures of the test and save this information in the internal memory of the device. At the beginning, the app allows for selecting the exercises to be administered from a list and then launches the exercises sequentially, in the same order as they are described below.

#### Tapping

**Fig. 1:**
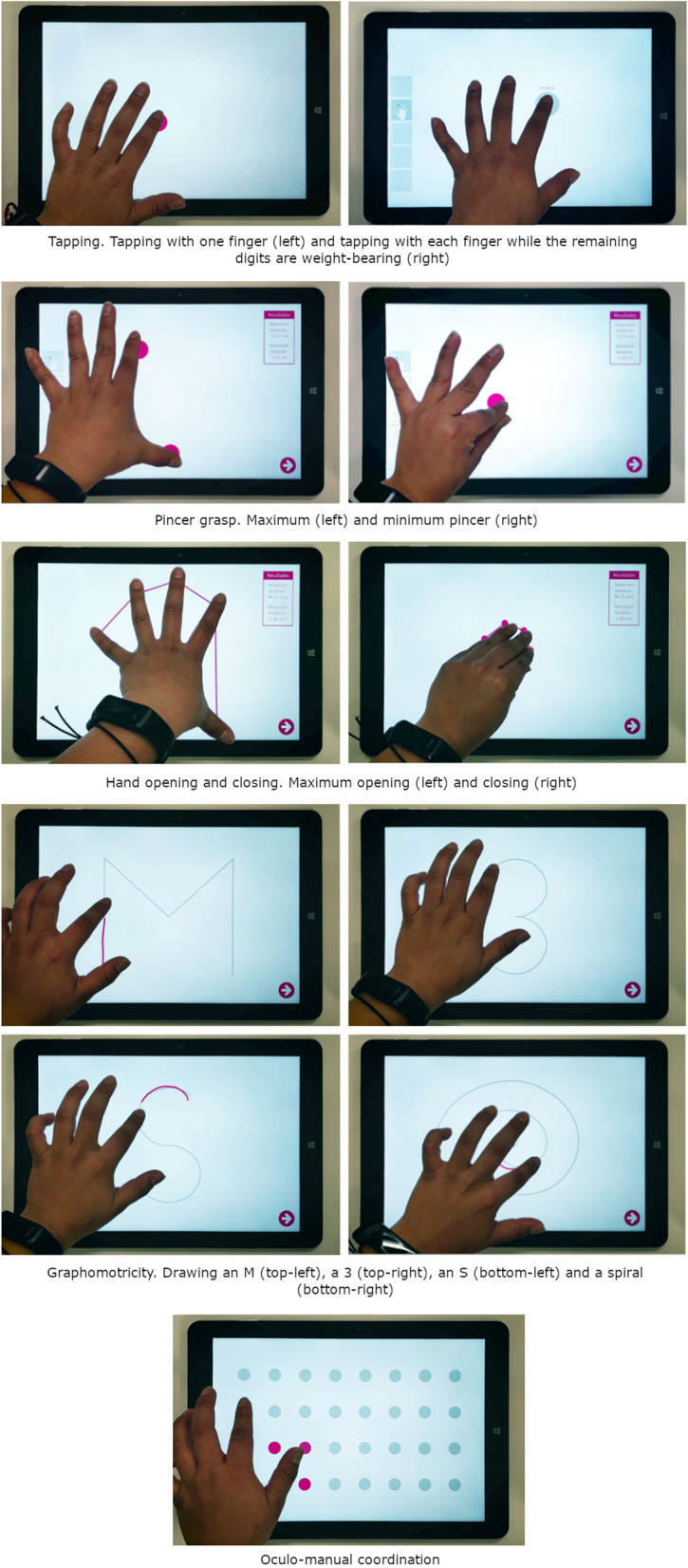
Interaction with the exercises of the Hand Assessment Test. Interaction with all the exercises of the *Hand Assessment Test*. The pictures are taken from above

This exercise assesses finger mobility and control. The objective of the exercise is to tap on the screen with one finger as many times as possible in 10 s (Fig. 1). This exercise is commonly performed with the index finger, as it is usually the most dexterous and sensitive finger. In case of loss, motor or sensory impairment, any other finger can be used, as the app does not identify the finger being used. During the testing, the exercise provides feedback of the contact point of the fingers with the screen (pink). The outcome measure of this exercise is the number of finger touches.

#### Tapping with each finger while the remaining digits are weight-bearing

This exercise assesses finger mobility, hand dexterity and coordination. The objective of the exercise is to tap on a calibrated spot of the screen with one finger while maintaining the other fingers in contact with the screen as many times as possible in 10 s (Fig. 1). If touches are not performed within the spot or others fingers are not in contact with the screen, the number of touches are not taken into account. During the testing, the exercise provides feedback of the calibrated position of the finger (gray) and the contact point of the fingers with the screen (pink). The outcome measure of this exercise is the number of finger touches for each finger.

#### Pincer grasp

This exercise assesses finger mobility, dexterity and coordination. The objective of the exercise is to move away one finger as much as possible from the thumb (extension of the metacarpophalangeal and interphalangeal joints) and move it as close as possible towards it (flexion of the metacarpophalangeal and interphalangeal joints) (Fig. 1). During the testing, the exercise provides feedback of the contact point of the fingers with the screen. The outcome measures of the exercise are the maximum and minimum distance reached for each finger.

#### Hand opening and closing

This exercise assesses hand mobility and coordination. The objective of the exercise is to open and close the hand as much as possible, by extending and flexing the fingers maintaining them in contact with the screen (Fig. 1). During the testing, the exercise provides feedback of the contact point of the fingers with the screen and straight lines connecting the contact points (pink). The outcome measures of the exercise are the maximum and minimum area of the hand (see Additional file 1).

#### Graphomotricity

This exercise assesses arm and hand coordination. The objective of the exercise is to trace the lines of four different figures over lightened versions of them (Fig. 1). Figures to draw are “M”, “3”, “S” and a spiral. During the testing, the exercise provides feedback of the lightened figures (gray) and traced lines (pink). The outcome measure of the exercise is the dissimilarity between the figure showed and the participants’ trace (see Additional file 1).

#### Oculo-manual coordination

This exercise assesses arm, hand and finger coordination, and finger dexterity. The objective of the exercise is to touch 32 visual targets arranged on a 4 × 8 grid as fast as possible (Fig. 1). The targets are always visible and can be touched in any order. During the testing, the exercise provides feedback of the targets (gray), highlighting those that were already touched (pink). The outcome measure of the exercise is the time to touch all the targets.

### Procedure

**Fig. 2:**
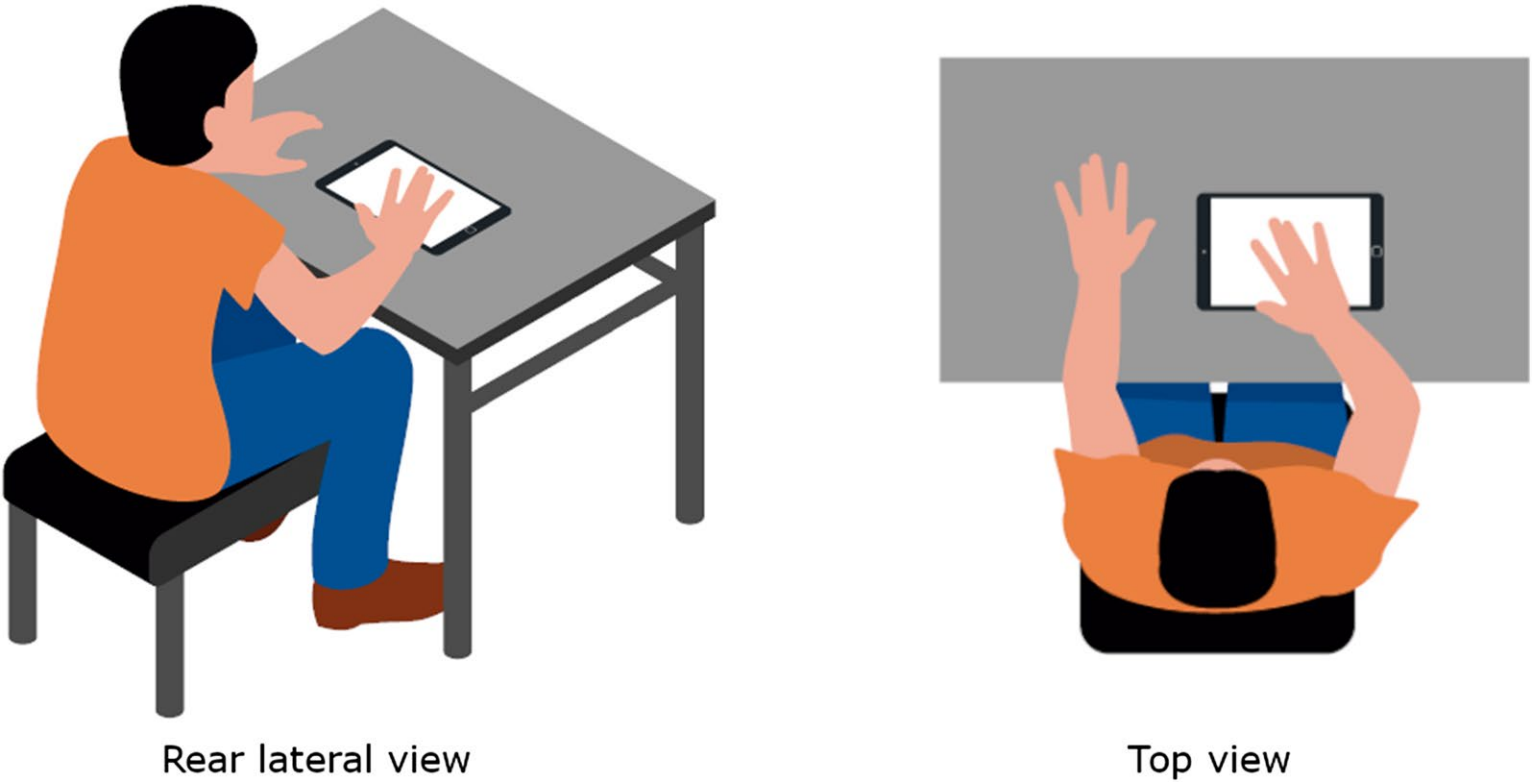
Experimental setup. Participants sat in a chair without armrests or back support, with their arms on a table, upon which a 12-inch tablet was located. The tablet lied flat on the table with the screen pointing upwards at a comfortably reachable distance by the participants. The participants completed all the exercises that they were capable of with their more affected arm

An experimenter conducted all the sessions. Participants were briefly introduced to the objectives of the study and were asked to sit in a comfortable position, in a chair without armrests or back support, in front of a table (Fig. 2). Participants who required a wheelchair were allowed to remain in the wheelchair, but the armrests were removed. Participants were invited to approach the table and lay their arms on its surface. The arms of the participants were not fixed. The experimenter placed the tablet lying flat on the table with the screen pointing upwards at a comfortably reachable distance by the participant. Before each exercise, the experimenter corrected the position and orientation of the tablet and the participants’ arm and posture if needed. The experimenter explained and performed each exercise to the participants before their attempt. No practice trials were allowed. The experimenter started each exercise by pressing a button and supervised the performance. In case of an adverse event in the performance of the tablet or the participant, the trial was discarded and the exercise was repeated. If the trial was successful, the experimented prepared the next exercise. Participants were allowed to rest between exercises on demand.

All participants were assessed with all of the *Hand Assessment Test* subtests, in the predefined and fixed order of the app, described in the previous subsection. The participants completed all the exercises that they were capable of with their more affected arm once but for the *Oculo-manual Coordination Test*, which was performed three times using (a) the index finger; (b) a touchscreen pen (or stylus) with the shape of a conventional pen; and (c) the same touchscreen pen, with the added requirement that the pen must be dropped and grasped between touches. Conditions that involved interacting with the pen were included to investigate possible different effects derived from the increased difficulty that represent handling this instrument during writing tasks. Those tests that allowed assessing each finger separately were performed once with each finger, when possible. The experimenter skipped those trials that were not achievable by the participants. The assessment with the *Oculo-manual Coordination Test* took 15–20 min, depending on the condition of each participant.

Participants were also assessed using the *Fugl-Meyer Assessment for Upper Extremity* [41], the *Jebsen-Taylor Hand Function Test* [15], without considering the “moving heavy objects” item of the scale, the *Box and Block Test* [8], and the *Nine Hole Peg Test* [16]. The assessment with the clinical scales and tests took around 45 min, depending on the condition of each participant.

From the total sample of participants, a minimum of 22 subjects (25% of the minimum required sample size) were assessed three times using the *Hand Assessment Test*. These participants were assessed twice by the same experimenter, to determine the intra-rater reliability, and a third time by a separate experimenter, to determine the inter-rater reliability. The participants received the same instructions in all the assessments.

All measurements were conducted on the same day, allowing participants to rest between assessments.

### Data analysis

Statistical data analysis was conducted using SPSS v22 (Inc., Chicago, IL, USA). The normality of the data distribution was analyzed with the Shapiro–Wilk test, and homoscedasticity was analyzed with the Levene test.

Data analysis performed as described in similar studies [42, 43]. First, the convergent validity of the *Hand Assessment Test*, compared with clinical tools, was investigated using Pearson’s correlation analysis. Second, the inter and intra-rater reliabilities of the app were determined, using a two-way, random-effects model intra-class correlation coefficient (ICC), with a single rate/measurement (2,1). Correlations greater than 0.8 were considered to be excellent. Values ranging from 0.6 to 0.8 and from 0.4 to 0.6 were considered to be indicators of strong and moderate reliability, respectively. Values ranging from 0.2 to 0.4 and below 0.2 were considered to be indicators of weak and very-weak reliability, respectively [44]. The standard error of measurement and the minimal detectable change were also obtained [45]. The standard error of measurement describes the effect of measurement error on individual results in a test. In other words, it quantifies the precision of individual results on a test. It can be interpreted as an assessment of reliability within individual results. The minimal detectable change, also referred to as the smallest detectable difference or sensitivity to change, is a statistical estimate of the smallest amount of change that can be detected by a measure that corresponds to a noticeable change in its measurements. In other words, it is the minimal amount of change of a measurement to be greater than the within subject variability and measurement error. In this study, the estimations of the minimal detectable change were based on a 95% confidence interval. Finally, although convergent validity showed the extent to which the app and clinical assessment tools of motor impairment were related, an additional analysis was conducted to compare how subjects with mild and moderate impairments performed in the app. The ability of the app to identify motor impairment severity was explored using Student’s t-tests. Specifically, participants were classified into two groups, according to the severity of their motor impairments. A moderately severe group, defined as having scores between 19 and 46 on the *Fugl-Meyer Assessment for Upper Extremity*, and a mildly severe group, defined as having scores higher than 47 on the same scale [46]. The maximum type I error was established at 5%.

Prior to performing any analyses, outliers were identified, as those data points with z-scores higher than three-fold the standard deviation, and consequently removed. Participants who were considered outliers in more than two measures were removed from analyses.

Although different analyses were performed for each subtest, their results are grouped according to their functional implications into the following five categories: (1) *Tapping*, which includes the *Tapping* test and *Tapping with each finger while the remaining digits are weight-bearing*; (2) *Finger flexion*, which includes the minimum value of the *Pincer grasp* with each finger and the minimum hand area during the *Hand Opening and Closing* test; (3) *Finger extension*, which (opposite to the previous category) includes the maximum value of the *Pincer grasp* with each finger and the maximum hand area during the *Hand Opening and Closing* test; (4) *Graphomotricity*, which includes the scores of the four subtests (drawing an “M”, a “3”, an “S”, and a spiral); and (5) *Oculo-manual coordination*, which includes the scores in the homonymous test, under the three different conditions described in the procedure (using a finger, a pen, and a pen that must be dropped and grasped after each touch).

## Results

### Participants

Ninety-six individuals with stroke were enrolled in this study, 8 of whom were classified as outliers and were excluded from the analysis. A final sample of 88 participants, 30 women and 58 men, were included in the study (Table 1). The participants had a mean (SD) age of 57.27 (12.75) years and a mean time since injury of 49.13 (59.68) months. Participants had suffered either an ischemic (n = 47) or hemorrhagic (n = 41) stroke, which affected either the left (n = 47) or right hemisphere (n = 35) or had equivalent bilateral effects (n = 6). No statistical differences between groups of different impairment severity emerged in any variable but in the score on the *Fugl-Meyer Assessment for Upper Extremity.* Twenty-three of these participants were additionally considered for the reliability analysis.

**Table 1:** Demographic and clinical characteristics of the participants

Variable	All participants (n = 88)	Participants with mild impairment (n = 69)	Participants with moderate impairment (n = 19)	Significance
*Age (years)*	57.27 (12.75)	57.39 (11.59)	56.84 (16.68)	*p* = .870
*Sex (n, %)*				*p* = .405
Women	30 (34.1%)	22 (31.9%)	8 (42.1%)	
Men	58 (65.9%)	47 (68.1%)	11 (57.9%)	
Time since injury (months)	49.13 (59.68)	46.15 (52.53)	59.96 (47.82)	*p* = .375
*Etiology (n, %)*				*p* = .580
Ischemic	46 (52.3%)	35 (50.7%)	11 (57.9%)	
Hemorrhagic	42 (47.7%)	34 (49.3%)	8 (42.1%)	
*Oxford classification (n, %)*				*p* = .453
TACI	3 (3.4%)	2 (5.7%)	1 (9.1%)	
PACI	29 (33.%)	23 (65.7%)	6 (54.5%)	
LACI	6 (6.8%)	3 (8.6%)	3 (27.3%)	
POCI	8 (9.1%)	7 (20.0%)	1 (9.1%)	
*Lesion side (n, %)*				*p* = .892
Left	47 (53.4%)	36 (52.2%)	11 (57.9%)	
Right	35 (39.8%)	28 (40.6%)	7 (36.8%)	
Bilateral	6 (6.8%)	5 (7.2)	1 (5.3%)	
Fugl-Meyer Assessment for Upper Extremity	53.02 (9.52)	57.10 (5.28)	38.21 (6.16)	*p* < .001

Data are expressed in mean (standard deviation)

### Convergent validity

**Fig. 3:**
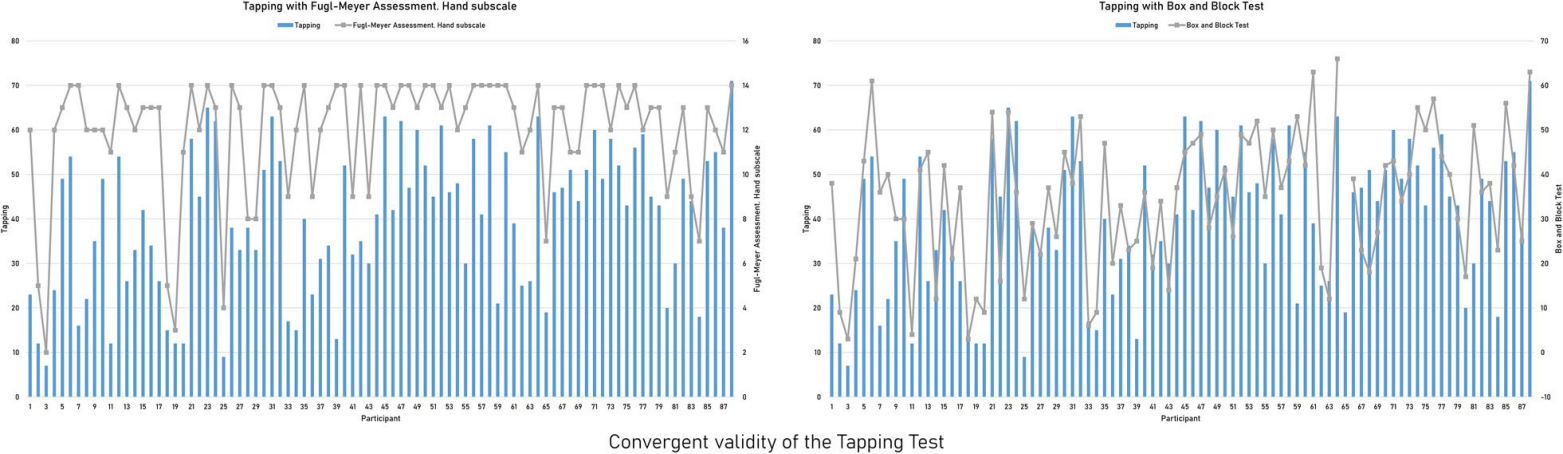
Individual performance on the Tapping test and the clinical scales with the highest convergent validity. Individual performance of all the participants on the *Tapping* test and on the hand subscale of the *Fugl-Meyer Assessment* and the *Box and Block Test*, which showed a convergent validity of .64 (*p* < .01) and .70 (*p* < .01), respectively

**Fig. 4:**
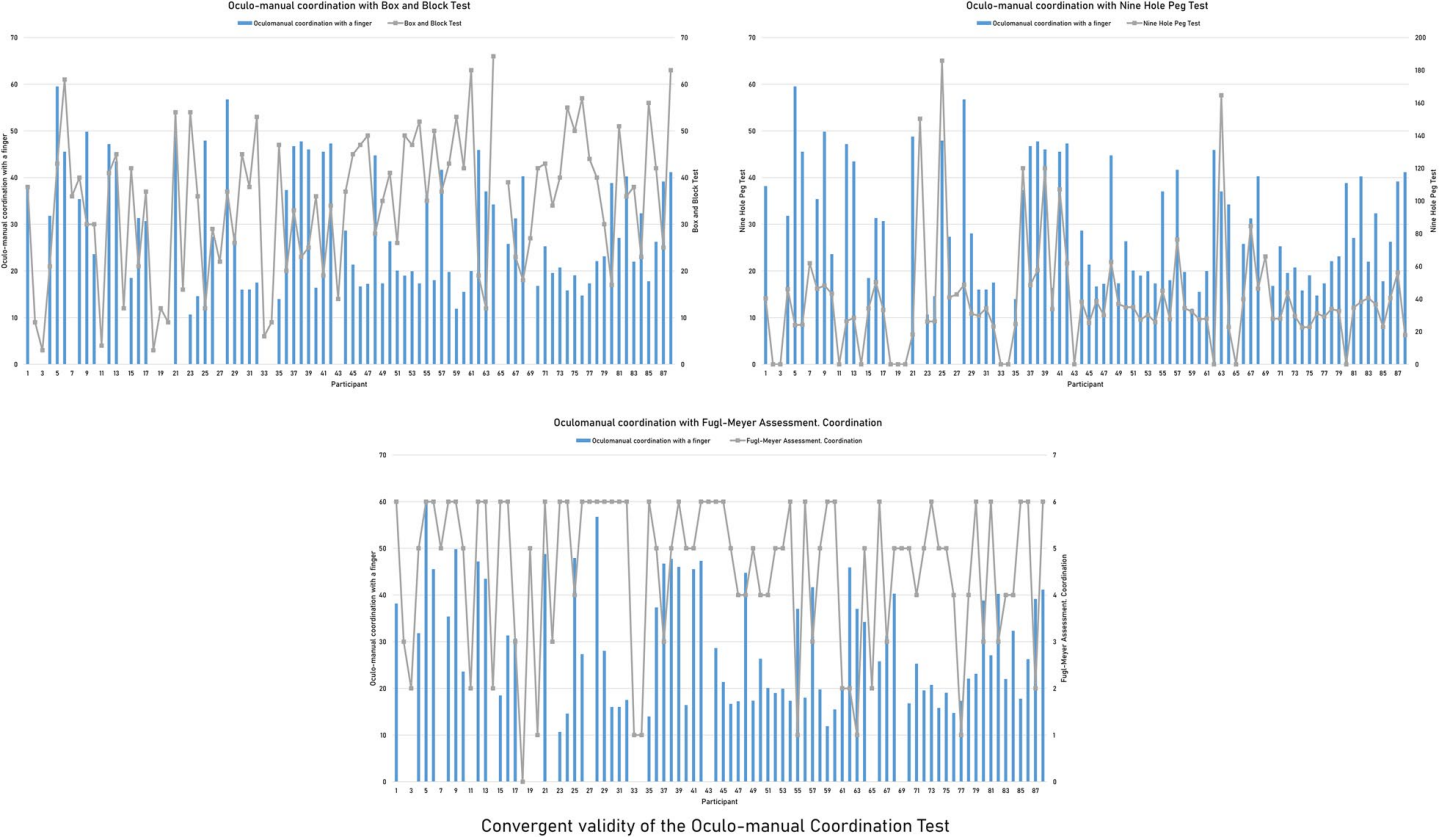
Individual performance on the Oculo-manual coordination test and the clinical scales with the highest convergent validity. Individual performance of all the participants on the *Oculo-manual coordination test* with a finger and on the *Box and Block Test,* the *Nine Hole Peg Test*, and the coordination subscale of the *Fugl-Meyer Assessment*, which showed a convergent validity of − .70 (*p* < .01), .71 (*p* < .01) and − .60 (*p* < .01), respectively

All interactions with the exercises in the app and the clinical tests are provided in Additional file 2. All exercises included in the *Tapping* category showed significant correlations of variable strength, which were predominantly moderate or strong, with almost all of the clinical tools with similar motor requirements (Table 2). The distribution of the individual performance in the *Tapping* test and in the clinical instruments that evidenced the highest convergent validity are shown in Fig. 3. Poorer correlations, moderate at best, were found between exercises included in the *Finger flexion* category and clinical instruments, which appeared to better match the required motor skills (Table 3). The writing subtest of the *Jebsen-Taylor Hand Function Test* did not show any significant correlations with any measures included in the multi-touch exercises. Similarly, the hand closing area also failed to show significant correlations with any clinical measures. The exercises included in the *Finger extension* category showed moderate correlations with almost all comparable clinical measurements (Table 4). Moderate correlations were also found between the exercises included in the *Graphomotricity* category and the clinical test measurements, except for the writing subtest of the *Jebsen-Taylor Hand Function Test* (Table 5). The performances in the *Oculo-manual coordination test,* under different conditions, showed prevalent moderate to strong correlations, which had the highest values when no external object was manipulated (Table 6). The distribution of the individual performance in this test and in the clinical instruments that evidenced the highest convergent validity are shown in Fig. 4.

**Table 2:** Convergent validity of the tapping exercises with clinical instruments

	Fugl-Meyer Assessment Scale		Jebsen-Taylor Hand Function Test			Box and Block Test	Nine Hole Peg Test
	Hand	Coordination	Card turning	Picking up small common objects	Stacking checkers		
Tapping	.64**	.42**	− .62**	− .52**	− .57**	.70**	− .53**
Tapping with the thumb while the remaining digits are weight-bearing	.52**	.28*	− .59**	− .57**	− .51**	.69**	− .53**
Tapping with the index finger while the remaining digits are weight-bearing	.51**	.25*	− .59**	− .60**	− .57**	.66**	− .59**
Tapping with the middle finger while the remaining digits are weight-bearing	.46**	.29*	− .57**	− .62**	− .45^**^	.63^**^	− .48^**^
Tapping with the ring finger while the remaining digits are weight-bearing	.54**	.46**	− .53**	− .59**	− .34**	.55**	− .45**
Tapping with the little finger while the remaining digits are weight-bearing	.43**	.36**	− .49**	− .61**	− .42**	.64**	− .42**

Moderate or stronger correlations are highlighted in bold

*p < 0.05; **p < 0.01

**Table 3:** Convergent validity of the finger flexion exercises with clinical instruments

	Fugl-Meyer Assessment Scale	Jebsen-Taylor Hand Function Test					Box and Block Test	Nine Hole Peg Test
	Hand	Writing	Card turning	Picking up small common objects	Feeding	Stacking checkers		
Minimum index finger pincer grasp	− .42**	.07	.05	.00	.20	.25*	− .30*	.19
Minimum middle finger pincer grasp	− .48**	.14	.35**	.35**	.32*	.64**	− .40**	.53**
Minimum ring finger pincer grasp	− .38**	.15	.32*	.31*	.42**	.41**	− .43**	.29*
Minimum little finger pincer grasp	− .28*	.03	**.**37**	.43**	.59**	.27*	− .54**	.23
Hand closing area	− .01	.18	.16	.10	− .07	.25	− .14	.18

Moderate or stronger correlations are highlighted in bold

*p < 0.05, **p < 0.01

**Table 4:** Convergent validity of the finger extension exercises with clinical instruments

	Fugl-Meyer Assessment Scale	Jebsen-Taylor Hand Function Test
	Hand	Moving light objects
Maximum index finger pincer grasp	.56**	− .50**
Maximum middle finger pincer grasp	.44**	− .52**
Maximum ring finger pincer grasp	.45**	− .49**
Maximum little finger pincer grasp	.36**	− .50**
Hand opening area	.58**	− .46**

Moderate or stronger correlations are highlighted in bold

*p < 0.05; **p < 0.01

**Table 5:** Convergent validity of the graphomotricity exercises with clinical instruments

	Fugl-Meyer Assessment Scale	Jebsen-Taylor Hand Function Test			Box and Block Test	Nine Hole Peg Test
	Coordination	Writing	Picking up small common objects	Stacking checkers		
Drawing an “M”	− .42**	.19	.46**	.69**	− .42**	.62**
Drawing an “S”	− .41**	.15	.34*	.49**	− .37*	.40**
Drawing a “3”	− .47**	.19	.48**	.55**	− .43**	.58**
Drawing a spiral	− .34*	.24	.27	.48**	− .33*	.35*

Moderate or stronger correlations are highlighted in bold

*p < 0.05; **p < 0.01

**Table 6:** Convergent validity of the oculo-manual coordination exercises with clinical instruments

	Fugl-Meyer Assessment Scale		Jebsen-Taylor Hand Function Test		Box and Block Test	Nine Hole Peg Test
	Hand	Coordination	Feeding	Picking up small common objects		
Coordination with a finger	− .44**	− .60**	.70**	.65**	− .70**	.71**
Coordination with a pen	− .34**	− .27*	.46**	.41**	− .50**	.37**
Coordination with a pen involving dropping and grasping	− .34**	− .11	.36**	.46**	− .40**	.42**

Moderate or stronger correlations are highlighted in bold

*p < 0.05; **p < 0.01

The signs for all of the correlations between the measurements made using the app and those made using clinical tests supported the consistency of the instrumented measurements, as better performances on these tests were associated with better performances as measured by the clinical tools. As an example, higher numbers of finger taps were associated with more blocks in the *Box and Block Test* and less time required to complete the *Nine Hole Peg Test*, and a larger hand opening area was associated with a higher score on the *Fugl-Meyer Assessment for Upper Extremity.*

### Inter- and intra-rater reliability, standard error of measurement, and minimal detectable change

Moderate to excellent reliability was found for all measurements made using the app (Table 7).

**Table 7:** Reliability of the instrumented exercises with clinical instruments

	Intra-rater reliability	Inter-rater reliability	Standard error of measurement	Minimal detectable change
Tapping (n)	.90**	.95**	.49	1.38
Tapping with the thumb while the remaining digits are weight-bearing (n)	.85**	.83**	1.30	3.62
Tapping with the index finger while the remaining digits are weight-bearing (n)	.90**	.87**	.97	2.69
Tapping with the middle finger while the remaining digits are weight-bearing (n)	.82**	.86**	1.45	4.00
Tapping with the ring finger while the remaining digits are weight-bearing (n)	.84**	.63*	1.39	3.85
Tapping with the little finger while the remaining digits are weight-bearing (n)	.90**	.82**	.65	1.79
Minimum pincer grasp with the index finger (cm)	.70**	.85**	.20	.54
Maximum pincer grasp with the index finger (cm)	.57*	.53*	.52	1.45
Minimum pincer grasp with the middle finger (cm)	.63**	.41*	.28	.77
Maximum pincer grasp with the middle finger (cm)	.77**	.80**	.35	.98
Minimum pincer grasp with the ring finger (cm)	.69**	.86**	.17	.47
Maximum pincer grasp with the ring finger (cm)	.68**	.57*	.42	1.16
Minimum pincer grasp with the little finger (cm)	.75**	.71**	.17	.47
Maximum pincer grasp with the little finger (cm)	.77**	.59*	.31	.84
Hand closing area (cm^2^)	.57*	.96**	.26	.71
Hand opening area (cm^2^)	.88**	.79**	.23	.63
Drawing an “M” (cm)	.60*	.75**	9.01	25.01
Drawing an “S” (cm)	.90**	.92**	2.09	5.79
Drawing a “3” (cm)	.94**	.83**	1.86	5.15
Drawing a spiral (cm)	.85**	.71**	10.58	29.33
Coordination with a finger (s)	.96**	.92*	.83	2.30
Coordination with a pen (s)	.53*	.83**	3.59	9.96
Coordination with a pen involving dropping and grasping (s)	.91**	.68*	2.15	5.96

Moderate or stronger correlations are highlighted in bold

*p < 0.05; **p < 0.01

The tapping and coordination exercises performed with the index finger provided the best results among all the app measurements. The pincer grasp, in contrast, showed the poorest results. The exercises that required tracing an “S” had higher convergent validity and was more reliable than the rest of the graphomotricity exercises.

No systematic variation of the data that could reveal a consistent learning effect or a fatigue-related decrease in performance was detected during the multiple assessment.

### Sensitivity to the severity of motor impairments

Consistently better performances for all measurements made using the app were found among those participants with mild motor impairments, compared with moderately impaired participants (Table 8). However, significant differences between groups were almost exclusively found for exercises that involved tapping and maximum finger pincer grasp.

**Table 8:** Sensitivity to motor impairment severity

	Mild motor impairment		Moderate motor impairment	
	Number of participants	Performance	Number of participants	Performance
Tapping (n)	69	43.97 (14.47)	19	25.26 (13.51)**
Tapping with the thumb while the remaining digits are weight-bearing (n)	62	34,44 (11,46)	14	17,71 (11,08)**
Tapping with the index finger while the remaining digits are weight-bearing (n)	63	37,65 (12,43)	13	18,31 (11.21)**
Tapping with the middle finger while the remaining digits are weight-bearing (n)	61	34.34 (11.24)	12	17.50 (9.29)**
Tapping with the ring finger while the remaining digits are weight-bearing (n)	51	28.18 (9.10)	10	12.00 (9.01)**
Tapping with the little finger while the remaining digits are weight-bearing (n)	57	28.86 (9.92)	12	13.50 (8.50)**
Minimum index finger pincer grasp (cm)	56	1.32 (0.70)	12	1.73 (1.20)
Maximum index finger pincer grasp (cm)	66	13.05 (2.04)	15	10.31 (2.29)**
Minimum middle finger pincer grasp (cm)	55	1.25 (0.74)	11	1.59 (0.71)
Maximum middle finger pincer grasp (cm)	65	17.94 (2.28)	14	12.39 (2.48)**
Minimum ring finger pincer grasp (cm)	53	1.17 (0.85)	9	2.12 (1.43)
Maximum ring finger pincer grasp (cm)	64	15.81 (2.35)	12	12.13 (2.49)**
Minimum little finger pincer grasp (cm)	53	1.39 (0.92)	9	2.128 (0.953)*
Maximum little finger pincer grasp (cm)	64	15.59 (2.87)	12	12.69 (4.24)*
Hand closing area (cm^2^)	56	2.48 (0.73)	16	2.47 (0.54)
Hand opening area (cm^2^)	67	95.47 (23.84)	17	67.88 (25.60)**
Drawing an “M” (cm)	36	58.25 (34.76)	7	82.57 (37.71)
Drawing an “S” (cm)	36	33.82 (16.62)	7	40.01 (9.92)
Drawing a “3” (cm)	36	34.19 (21.41)	7	52.09 (23.39)
Drawing a spiral (cm)	36	129.30 (77.22)	7	145.26 (64.58)
Coordination with a finger (s)	63	28.37 (12.60)	10	35.87 (10.58)
Coordination with a pen (s)	63	29.79 (11.91)	11	39.45 (11.18)*
Coordination with a pen involving dropping and grasping (s)	39	80.52 (32.34)	3	163.07 (87.81)

Data are expressed in mean (standard deviation)

*p < 0.05; **p < 0.01

## Discussion

This study investigated the convergent validity, reliability, and sensitivity of a dedicated app, designed to examine hand mobility and dexterity in a sample of individuals with stroke. The app showed coherent significant correlations of variable strengths (which were predominantly moderate) with different clinical scales and tests. The highest convergent validity values were found for exercises that involved tapping and oculo-manual coordination. Moderate to excellent reliability was found for all measurements made by the instrumented assessment. Furthermore, exercises that involved tapping and maximum pincer grasp showed the highest sensitivity for distinguishing mild to moderate motor impairment.

The consistent correlations found between exercises in the *Tapping* category and all clinical instruments, could indicate the clinical implications of finger tapping. A previous study also reported a significant interaction between finger tapping assessments and another clinical instrument [47]. Interestingly, although the recovery of the tapping rate and the independence of finger movements appear to be more difficult than the recovery of gross hand motor impairment [48], the clinical relevance of these tests should be considered because finger-tapping has been shown to be a predictor of functional outcomes after stroke [49]. The correlation between the tapping test and the *Box and Block Test*, which exhibited the strongest interaction, is supported by a preliminary proof of concept study [2]. The weak correlations found between exercises that involved tapping and the coordination subscale of the *Fugl-Meyer Assessment for Upper Extremity* may be due to this scale involving the coordination of the whole arm and not only the hand. The moderate to strong correlations found between the tapping exercises and the remaining clinical tools could indicate that tapping exercises have the potential to reflect hand functionality, especially finger mobility and coordination. Although excellent agreement was found between assessments and experimenters, the flexion and extension of the ring finger showed remarkably different inter and intra-rater variability. The particular muscular characteristics and cortical representation of the fingers could explain why the thumb, index, and middle fingers are generally easier to control and are more independent than the ring finger [50], which may explain the reliability discrepancies observed for measurements of ring finger mobility. Previous research has shown that finger-tapping was able to successfully discriminate between healthy individuals and individuals with stroke [49]. The sensitivity of the app could go beyond this, as the exercises that involve tapping were effectively able to discriminate between different levels of motor impairment severity after stroke.

For the *Finger flexion* and *Finger extension* categories, the absence of any relation between the minimum pincer grasp with the index finger and either the *Nine Hole Peg Test* or almost all of the subscales on the *Jebsen-Taylor Hand Function Test* and between the hand closing area and all clinical tests should be highlighted. Although the pincer grasp and hand closing have direct and important relevance for object manipulation and functional tasks, they showed weaker convergent validity values with clinical scales than the pincer grasp with the other fingers, which presumably have fewer functional implications. The hand positioning during the test, which was freely chosen by participants, according to their capacity, may have caused these weak correlations. In cases of complete pronation of the hand during the testing procedures, contact with the screen could have been affected by the nails, with more intensity in the maximum flexion of the index finger (minimum pincer grasp and hand closing), leading to incorrect measurements. The (predominantly) moderate correlations that were consistently found between exercises in the *Finger extension* category, where the nail was less likely to interfere, and the clinical tests suggested that the performances of these exercises were less affected than the performances of those exercises that required finger flexion, which also supports the potential nail interference as a possible explanation for the misleading results. Slight supination of the hand could have facilitated direct contact between the lateral side of the fingers with the screen of the tablet in the *Pincer grasp* tests, and should be considered in further use of the app However, as stroke can cause severe dysfunctions in wrist functions, even in cases of mild impairment [51], wrist rotation can be challenging for many individuals; therefore, interactions with this test should be performed with caution. Consequently, finger grasp examination using this test should be restricted to those individuals capable of performing slight supination of the hand. The sensitivity of the exercises that involved finger extension, but not finger flexion, for detecting motor impairment severity could also highlight the difficulties of the app for registering finger flexion and target the nail interference as the cause. Future studies could also explore the possibility of using touchscreen gloves during the *Pincer Grasp* and *Hand Opening and Closing* tests. The variable reliability found for exercises in the *Finger flexion* and *Finger extension* categories, which ranged from moderate to excellent values, could also highlight the variable performance of the participants during the repeated assessments required for the inter- and intra-rater reliability. Future studies using this app should consider the averaged performance scores for these exercises across different trials, rather than only one attempt.

The moderate to strong correlations detected between the exercises in the *Graphomotricity* category and clinical instruments, except for the writing subtest of the *Jebsen-Taylor Hand Function Test*, could indicate that these exercises were especially able to reflect coordination, rather than writing skills. The absence of any correlations with the writing subtest of the *Jebsen-Taylor Hand Function Test* may be due to differences in the cognitive skills required by both instruments. Whereas the exercises used by the app only required the participants to trace the lines of four different figures over a lightened version of those figures, the subtest of the *Jebsen-Taylor Hand Function Test* required copying a 24-letter sentence and turning over 3″ × 5″ cards, which not only requires specific writing skills, such as planning, translating, and revising [52] but also working memory and metacognitive skills [53]. The excellent agreement between assessments and experimenters for those exercises that required drawing an “S” and a “3”, which was greater than the other exercises in this category, highlights the reliability of these subtests. However, although larger errors were associated with more severe motor impairments, the lack of statistical differences between individuals with mild and moderate functional deficits suggested that exercises that involve graphomotricity may not be responsive enough to detect specific dissimilarities in the performances of individuals with different motor conditions. A larger number of participants with moderate motor impairment would correct any imbalances between groups and prevent type II errors.

The convergent validity of the exercise associated with *Oculo-manual coordination* that required the coordination of the index finger supports its ability to reflect many of the skills required by some of the most widely used and recommended instruments [54]. The weaker correlations between the exercises in the category that involved grasping and those clinical instruments that also involved grasping could be due to the different types of grasping required. Whereas the exercise used for the app requested that a pen be grasped (not necessarily using a traditional tripod grasp), clinical instruments require different grasps to manipulate blocks and hold pegs, spoons, or sheets [55, 56]. The involvement of the pencil grasp during coordination exercises may have acted as an additional source of noise for the performance, as evidenced by the reduced reliability exhibited by these exercises compared with the non-use of a pen. The heterogeneity of the individual strategies used to compensate for motor limitations [57] and the difficulty adapting grip strength after stroke could also support these results [58]. Altered grip strength could also explain the worse results for participants with worse motor conditions during the test, as grip strength is not only strongly associated with sensorimotor function [59] but also can be a predictor of hand motor performance [60].

It is important to consider that, given the high accuracy of multitouch technology, the objective estimation of the outcome parameters of the exercises, and the consistency of the instructions given to the participants, the reliability results might mostly represent a variability in the participants’ performance, rather than any other factor. According to this, exercises that showed higher reliability, such as the tapping test, could be more robust against individual performance, and, conversely, exercises that showed lower reliability, such as the pincer grasps, could be more sensitive to this effect, which has been found in previous studies [42]. For this reason, it would be advisable that those exercises with poor reliability were assessed multiple times in future studies to counteract the effect of variable performance.

Although motor impairments can limit interactions with multi-touch devices [31], the results of our study supported the reported ability of individuals with stroke to interact with a tablet [2], which could be increased by using touchscreen gloves, and revealed the potential of this device to assess hand mobility, coordination, and function for this population. However, it is important to highlight that only individuals with moderate to mild impairments, such as those included in this study, might successfully interact with the assessing instrument. Generalization of the findings of our study and the ability to interact with the tests to severely impaired individuals is, therefore, unknown. Our findings showed variable convergent validity, reliability, and sensitivity to motor impairment among all of the exercises in the *Hand Assessment Test* and highlighted that those tests that involved tapping and maximum finger extension were optimal for assessing motor impairment. The results of these tests, along with the capacity of the app to provide unbiased and accurate measurements and the low costs and portability associated with common multi-touch devices, could support the use of these tests to assess hand mobility, coordination, and function after stroke not only in the clinical setting but also at home. Importantly, the tablet app could enable remote assessment of hand function, which could be especially relevant in cases of forced confinement during health crisis or discontinued treatment, and for individuals from rural and low-income urban areas with limited access to rehabilitation services. It should be also highlighted that the remote administration of rehabilitation services might result in a reduction of the transportation costs [61]. Future studies should pay particular attention to the interaction of the fingers with the multi-touch device and explore the possibility of including participants with more severe impairments and home-care programs.

## Conclusions

The *Hand Assessment Test* showed variable convergent validity with clinical scales and tests, reliability and sensitivity to motor impairment severity. Exercises that involved tapping and the maximum extension of the fingers exhibited the best properties, which could support their use as complementary approaches to the conventional clinical assessments of hand function after stroke.

## Supplementary Information

**Additional file 1:** Description of data: This file describes how the outcome measures of *Hand opening and closing* and *Graphomotricity* are estimated.

**Additional file 2:** Description of data: The table shows the convergent validity of all measures provided by the multi-touch app with all the clinical scales and tests under study.

## Data Availability

The datasets used and/or analysed during the current study are available from the corresponding author on reasonable request.

## Footnotes

1 The *Hand Assessment Test* app is available for Android and Windows devices. More information about the application can be found at www.nrhbgroup.com.
