# Supplementary material for: Validity, reliability, and sensitivity to motor impairment severity of a multi-touch app designed to assess hand mobility, coordination, and function after stroke
Source: J Neuroeng Rehabil. 2021 Apr 23;18:70. doi: 10.1186/s12984-021-00865-9 (PMC8066975; doi:10.1186/s12984-021-00865-9)
Supplement: Supplementary file 1 — Additional file 1: Description of data: This file describes how the outcome measures of Hand opening and closing and Graphomotricity are estimated. [file 12984_2021_865_MOESM1_ESM.pdf]

Additional file 1. Estimation of the outcome parameters of the *Hand opening and closing* and *Graphomotricity* exercises

Hand opening and closing

During this exercise, the contact points of the fingers with the multi-touch screen (x_i,y_i) are continuously registered. A polygon is defined at each frame considering the contact points as being the vertices of the polygon and its area is estimated using a shoelace algorithm implementation (1).

Analytically,

$$Area = \frac{1}{2}\left[\sum_{i=1}^{n} det \begin{vmatrix} x_i & x_{i+1} \\ y_i & y_{i+1} \end{vmatrix}\right] \text{cm}^2$$

Where, n is the number of sides of the polygon and (x_i, y_i) are the vertices of the polygon.

1. Younhee Lee and Woong Lim. *Shoelace formula: Connecting the area of a polygon and the vector cross product*. Mathematics teacher. 110 (8), 2017. 631-636. DOI: 10.5951/mathteacher.110.8.0631

Graphomotricity

For this exercise, the screen is divided into a 160x120 invisible grid. The positions of the constituent points of the different target figures ("M", "3", "S" and a spiral) in the grid were previously defined and are known. Two distance matrices, H and V, corresponding to the horizontal and vertical axes, were also previously defined by assigning a value to all the elements of the matrices corresponding to their distance to the nearest constituent point of the figure in the horizontal and vertical axes, respectively (Additional figure 1).

2	1	0	0
2	1	0	0
2	1	0	0
2	1	0	0

2	2	2	2
1	1	1	1
0	0	0	0
0	0	0	0

Additional figure 1. Example of the horizontal (left) and vertical (right) distance matrices. Constituent elements of the figures are highlighted in pink.

For each running device, the values of the elements of the distance matrices are translated into pixels taking into account the dimension of the screen of the device in pixels. Then, the pixel density (the number of pixels per inch) of the screen, also depending on each device, is used to estimate the physical distance of each element in centimeters. Finally, both distance matrices, H and V, are combined into a single distance matrix D, as follows:

$$D_{i,j} = \sqrt{({H_{i,j}}^2 + {V_{i,j}}^2)}$$

, where $D_{i,j}$, $H_{i,j}$, $V_{i,j}$ are the elements of the matrices.

During the exercise, the contact points of the interacting finger (or touchscreen pen) with the multi-touch screen (x, y) are registered. The error e is defined as the aggregated distance for all the contact points.

$$e = \sum_{i=1}^{N} D(x_i, y_i) \text{ cm}$$

, where e is the total error, D is the distance matrix, (x, y) are the coordinates of the contact points, and N is the total number of contact points made during the test.
