# Supplementary material for: Validity, reliability, and sensitivity to motor impairment severity of a multi-touch app designed to assess hand mobility, coordination, and function after stroke
Source: J Neuroeng Rehabil. 2021 Apr 23;18:70. doi: 10.1186/s12984-021-00865-9 (PMC8066975; doi:10.1186/s12984-021-00865-9)
Supplement: Supplementary file 2 — Additional file 2: Description of data: The table shows the convergent validity of all measures provided by the multi-touch app with all the clinical scales and tests under study. [file 12984_2021_865_MOESM2_ESM.pdf]

Additional file 2. Convergent validity of all measures of the *Hand Assessment Test* with clinical instruments

	Fugl-Meyer Assessment Scale					Jebsen-Taylor Hand Function Test							Box and Block Test	Nine Hole Peg Test
	Total score	Shoulder, elbox, forearm	Wrist	Hand	Coordination	Writing	Card turning	Picking up small common objects	Stacking checkers	Simulated feeding	Moving light objects	Moving heavy objects		
Tapping	**,55****	,32**	**,51****	**,64****	**,42****	-,31**	**-,62****	**-,52****	**-,57****	**-,49****	**-,54****	**-,40****	**,70****	**-,53****
Tapping with the thumb while the remaining digits are weight-bearing	**,51****	,36**	**,54****	**,52****	,28*	-,34**	**-,59****	**-,57****	**-,51****	**-,45****	**-,44****	**-,47****	**,69****	**-,53****
Tapping with the index finger while the remaining digits are weight-bearing	**,52****	,38**	**,56****	**,51****	,25*	**-,42****	**-,59****	**-,60****	**-,57****	**-,46****	**-,53****	**-,56****	**,66****	**-,59****
Tapping with the middle finger while the remaining digits are weight-bearing	**,56****	**,51****	**,50****	**,46****	,29*	-,37**	**-,54****	**-,62****	**-,45****	**-,51****	**-,49****	**-,54****	**,63****	**-,48****
Tapping with the ring finger while the remaining digits are weight-bearing	**,61****	**,52****	**,52****	**,54****	**,46****	**-,48****	**-,53****	**-,59****	-,34**	**-,42****	**-,53****	**-,55****	**,55****	**-,45****
Tapping with the little finger while the remaining digits are weight-bearing	**,59****	**,52****	**,56****	**,43****	,36**	-,32*	**-,49****	**-,61****	**-,42****	**-,54****	**-,40****	**-,44****	**,64****	**-,42****
Minimum pincer grasp with the index finger	-.23	-.08	-,24*	**-,42****	-.09	.071	.054	.00	,25*	.20	.14	-.04	**-,30***	.19
Maximum pincer grasp with the index finger	**,56****	**,46****	**,50****	**,56****	,26*	-,28*	**-,50****	**-,45****	**-,45****	**-,53****	**-,50****	-,30**	**,56****	**-,53****
Minimum pincer grasp with the middle finger	-.19	.01	-.23	**-,48****	-.07	.14	,35**	,35**	**,64****	,32*	,27*	.15	**-,40****	**,53****
Maximum pincer grasp with the middle finger	**,45****	,35**	**,51****	**,44****	.16	-.17	**-,45****	**-,40****	**-,52****	**-,41****	**-,52****	-,32**	**,54****	**-,43****

Minimum pincer grasp with the ring finger	-,36**	-,27*	**-,42****	-,38**	.02	.15	,32*	,31*	**,41****	**,42****	**,51****	.20	**-,43****	,29*
Maximum pincer grasp with the ring finger	**,46****	,37**	**,50****	**,45****	.13	-.14	**-,52****	**-,49****	**-,47****	**-,45****	**-,49****	-,36**	**,58****	-,39**
Minimum pincer grasp with the little finger	-,36**	-,27*	**-,41****	-,28*	-.15	.03	,37**	**,43****	,27*	**,59****	,30*	,26*	**-,54****	.23
Maximum pincer grasp with the little finger	,37**	, 26*	**,49****	**,34****	.09	-.16	**-,51****	**-,48****	**-,41****	-,29*	**-,50****	**-,40****	**,53****	-,37**
Hand closing area	.05	.08	-.06	-.01	.06	.18	.16	.10	.25	-.07	.09	.01	-.14	.18
Hand opening area	**,52****	,33**	**,45****	**,58****	,32**	-.10	**-,43****	-.22	-,34**	-.22	**-,46****	-.14	**,46****	-,26*
Drawing an "M"	-.24	-.14	-.29	-.035	**-,42****	.19	,36*	**,46****	**,69****	**,43****	**,44****	.27	**-,42****	**,62****
Drawing an "S"	-.21	-.10	-,34*	-.014	**-,41****	.15	.25	,34*	**,49****	,34*	.21	.22	-,37*	,40**
Drawing a "3"	-.24	-.14	-.22	-.069	**-,47****	.19	**,48****	**,48****	**,55****	.26	**,46****	.23	**-,43****	**,58****
Drawing a spiral	-.16	-.01	-,36*	-.013	-,34*	.24	.23	.27	**,48****	.17	.23	.14	-,31*	,35*
Coordination with a finger	**-,56****	**-,40****	**-,50****	**-,44****	**-,60****	**,41****	**,61****	**,67****	**,65****	**,70****	**,59****	**,65****	**-,70****	**,71****
Coordination with a pen	**-,41****	-,28*	**-,40****	-,34**	-,27*	.14	,37**	,40**	**,41****	**,46****	**,49****	,38**	**-,50****	**,37****
Coordination with a pen involving dropping and grasping	-.21	-.08	-.19	-,34**	-.11	-.06	,33**	**,42****	**,46****	,36**	**,42****	,37**	**-,40****	**,42****

*: $p < 0.05$. **: $p < 0.01$. Moderate or stronger correlations are highlighted in bold.
